# Biomimetic semiconducting polymer dots for highly specific NIR-II fluorescence imaging of glioma

**DOI:** 10.1016/j.mtbio.2022.100383

**Published:** 2022-08-07

**Authors:** Xiaoju Men, Xiaorui Geng, Zhe Zhang, Haobin Chen, Meng Du, Zhiyi Chen, Gang Liu, Changfeng Wu, Zhen Yuan

**Affiliations:** a Hunan Key Laboratory of the Research and Development of Novel Pharmaceutical Preparations, Academician Workstation, Changsha Medical University, Changsha, 410219, China; b Faculty of Health Sciences, University of Macau, Macau SAR, 999078, China; c Centre for Cognitive and Brain Sciences, University of Macau, Macau SAR, 999078, China; d Department of Biomedical Engineering, Southern University of Science and Technology, Shenzhen, Guangdong, China; e Department of Biomedical Engineering, School of Basic Medical Sciences, Central South University, Changsha, 410013, China; f Institute of Medical Imaging, Hengyang Medical School, University of South China, Hengyang, China; g Center for Molecular Imaging and Translational Medicine, Xiamen University, Xiamen, China

**Keywords:** Cell membrane coated nanoparticles, Second near-infrared imaging, Conjugated polymers, Polymer dots, Brain tumor

## Abstract

Glioma with very short medium survival time consists of 80% of primary malignant types of brain tumors. The unique microenvironment such as the existence of the blood-brain barrier (BBB) makes the glioma theranostics exhibit low sensitivity in diagnosis, a poor prognosis and low treatment efficacy. Therefore, the development of multifunctional nanoplatform that can cross BBB and target the glioma is essential for the high-sensitivity detection and ablation of cancer cells. In this study, C6 cell membrane-coated conjugated polymer dots (Pdots-C6) were constructed for targeted glioma tumor detection. As a new kind of biomimetic and biocompatible nanoprobes, Pdots-C6 preserve the complex biological functions of natural cell membranes while possessing physicochemical properties for NIR-II fluorescence imaging of glioma. After encapsulating C6 cell membrane on the surface of conjugated Pdots, Pdots-C6 exhibited the most favorable specific targeting capabilities in vitro and in vivo. In particular, this pilot study demonstrates that biomimetic nanoparticles offer a potential tool to enhance specific targeting to the brain, hence improving glioma tumor detection accuracy.

## Graphical abstract

**Figure:**
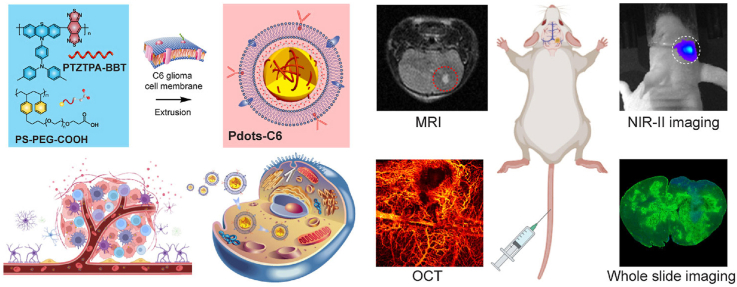

## 1 Introduction

Glioma is the most common category of brain tumors, whose median survival time is 12–15 months after diagnosis [[1], [2], [3]]. Magnetic resonance imaging (MRI) is routinely used for glioma detection [[4], [5], [6]]. However, the downside of MRI is that it is costly and time-consuming. By contrast, increasing attention has been paid to the development of fluorescence imaging techniques for cancer theranostics in the second near-infrared window (NIR-II, 1000–1700) [[7], [8], [9]]. Since the biological tissues show less autofluorescence in NIR-II window than that in the visible (400–700 ​nm) or the first near-infrared window (NIR-I, 700–900) [[10], [11], [12], [13]], NIR-II fluorescent probes can visualize the biological tissues with high temporal/spatial resolution and deep penetration [[14], [15], [16]]. However, most of the potential fluorescent probes do not exhibit a fluorescence signal in NIR-II window. Besides, it is also very challenging for the constructed nanoprobes to cross blood–brain barrier (BBB) and accumulate in the tumor site for enhanced glioma detection and therapy. Therefore, a facile nanoengineering modality is essential to endow NIR-II fluorescence nanoprobes with the BBB crossing ability and specific targeting effect.

In addition, nanoprobes can be designed through different strategies to increase the likelihood of crossing BBB, including direct BBB disruption, adsorptive-mediated transcytosis, cell-mediated transport, receptor-mediated transcytosis, carrier-mediated transcytosis, and reverse neuronal transport [17,18]. Meanwhile, the cell membrane-coated nanoparticles have recently been inspected for a variety of therapeutic and imaging applications [[19], [20], [21]], drug delivery [22], vaccination [23], and detoxification [24]. As a top-down coating approach, the use of biological materials is able to bestow synthesized nanoparticles with native cell functionalities [25]. In particular, by extracting the entire membrane from single cells and then linking cell membranes components to the surface of nanoprobes, all biologically relevant surface moieties are transferred and maintained for the biomimetic nanoplatform, such as their biocompatibility, retention of cellular properties, and adaptability [[26], [27], [28]]. For example, nanoparticles coating with red blood cell membrane can prolong their circulation time and reduce the accelerated blood clearance [29,30]. In addition, it was discovered that nanoprobes coated with active cell membranes can open a new avenue for passing BBB and targeting glioma [31,32]. Therefore, due to the preserved antigen and cell membrane structure, biomimetic nanoprobes can acquire special functions such as ligand recognition and targeting, long blood circulation and immune escape [33], providing a promising way for the diagnosis of brain tumors.

Further, multifunctional nanoprobes can serve as the contrast agents for high-sensitivity brain tumor detection [31,34]. In particular, semiconducting polymer dots (Pdots) have received extensive attention in biomedicine due to their outstanding biocompatibility, superior photostability, attractive optoelectronic properties, and abundant functional groups [[35], [36], [37]]. More importantly, luminescent conjugated polymers with NIR-II fluorescence properties were inspected, which were able to offer significant improvement for real-time imaging and glioma detection [[38], [39], [40]]. For example, Wu et al. developed NIR-II aggregation-induced emission (AIE) active Pdots with enhanced NIR-II fluorescence for through-skull mouse-brain imaging [41]. However, Pdots as exogenous materials, are easily identified by the immune system and quickly cleared by the liver and kidney before they can be transported into the brain [42]. Besides, pure Pdots have difficulties in crossing BBB, showing no specific targeting to brain tumor tissue. By contrast, nanoparticles with surface modification of BBB-targeting ligands or cell penetrating peptides have been constructed to cross BBB for highly accurate diagnosis of brain tumors [43,44]. More interestingly, the novel biomimetic nanoplatform exploiting natural cell membrane as the cloaks of nanoparticles, can be considered as a novel strategy for the design and synthesis of NIR-II fluorescence-based Pdots for glioma detection. Therefore, cell membrane components linked to the surface of Pdots might exhibit its unbeatable advantages in improving the outcomes of present glioma diagnosis protocols.

**Fig. 1:**
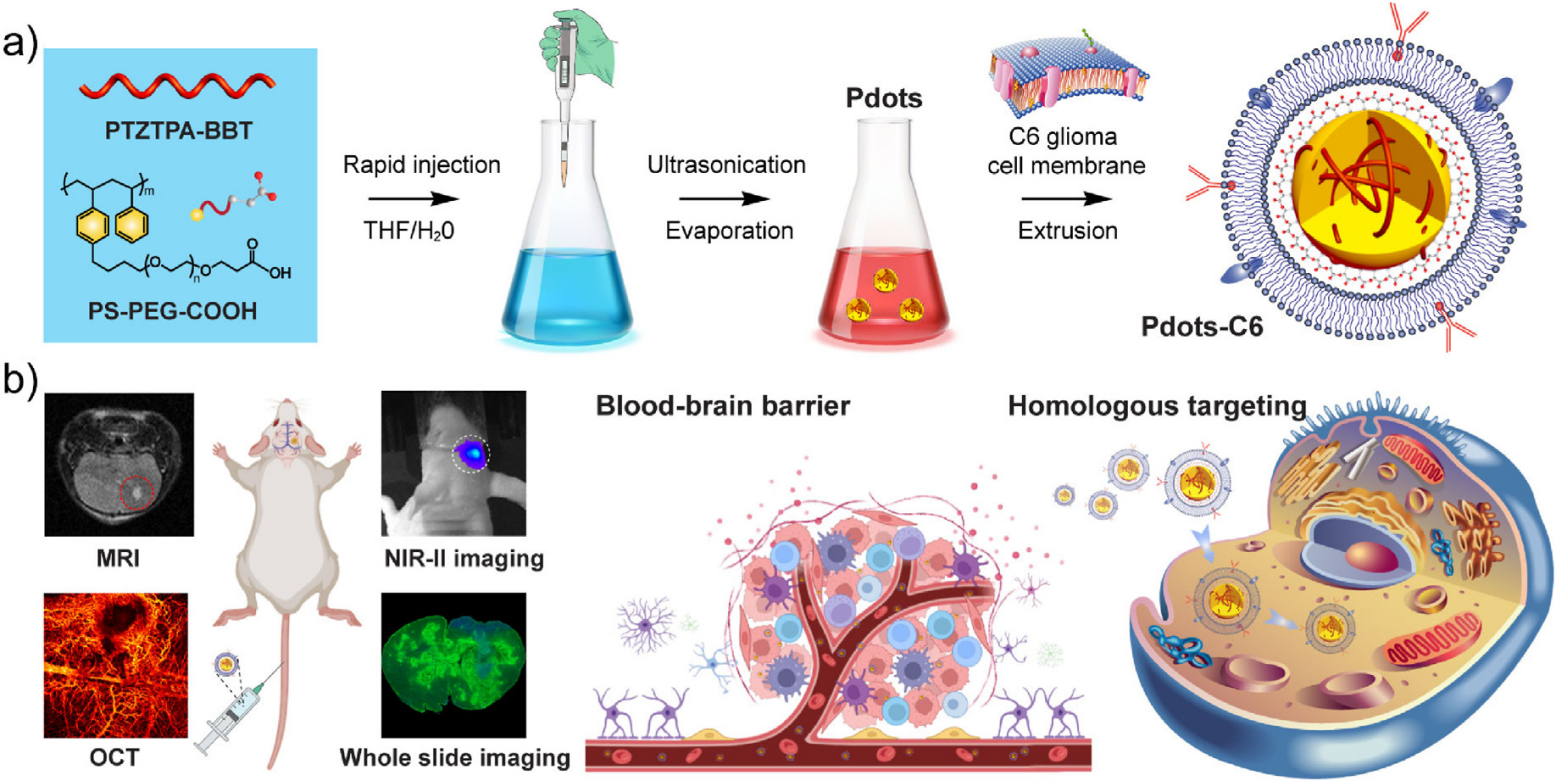
Schematic illustration of C6 glioma cell membrane coated Pdots (Pdots-C6) for targeted NIR-II fluorescence imaging of brain tumor. (a) Preparation process of Pdots-C6. (b) Schematic diagram of Pdots-C6 for crossing BBB and homologous targeting imaging.

In this study, we demonstrated that C6 glioma cell membrane-coated Pdots (Pdots-C6) as biomimetic biomaterials (Fig. 1a) for highly specific NIR-II fluorescence imaging of glioma. The homologous targeting, biodistribution, and NIR-II fluorescence imaging capability of Pdots-C6 were systematically accessed both in vitro and in vivo (Fig. 1b). Our findings illustrated that Pdots-C6 not only exhibited a homologous targeting effect at the cellular level but also specific targeting ability at the tissue level with high spatial resolution and deep penetration. As a new NIR-II fluorescence imaging strategy, Pdots-C6 hold great promise for clinical glioma detection as compared to pure Pdots due to the highly specific and crossing BBB abilities, excellent biocompatibility, and long circulation. The developed strategy opens the door to the development of customized biomimetic nanomaterials with different hybrid functions, which may be able to overcome the limitations of current nanomaterials-based imaging nanoplatforms.

## 2 Results and discussion

### 2.1 Design and synthesis of NIR-II PTZTPA-BBT

**Fig. 2:**
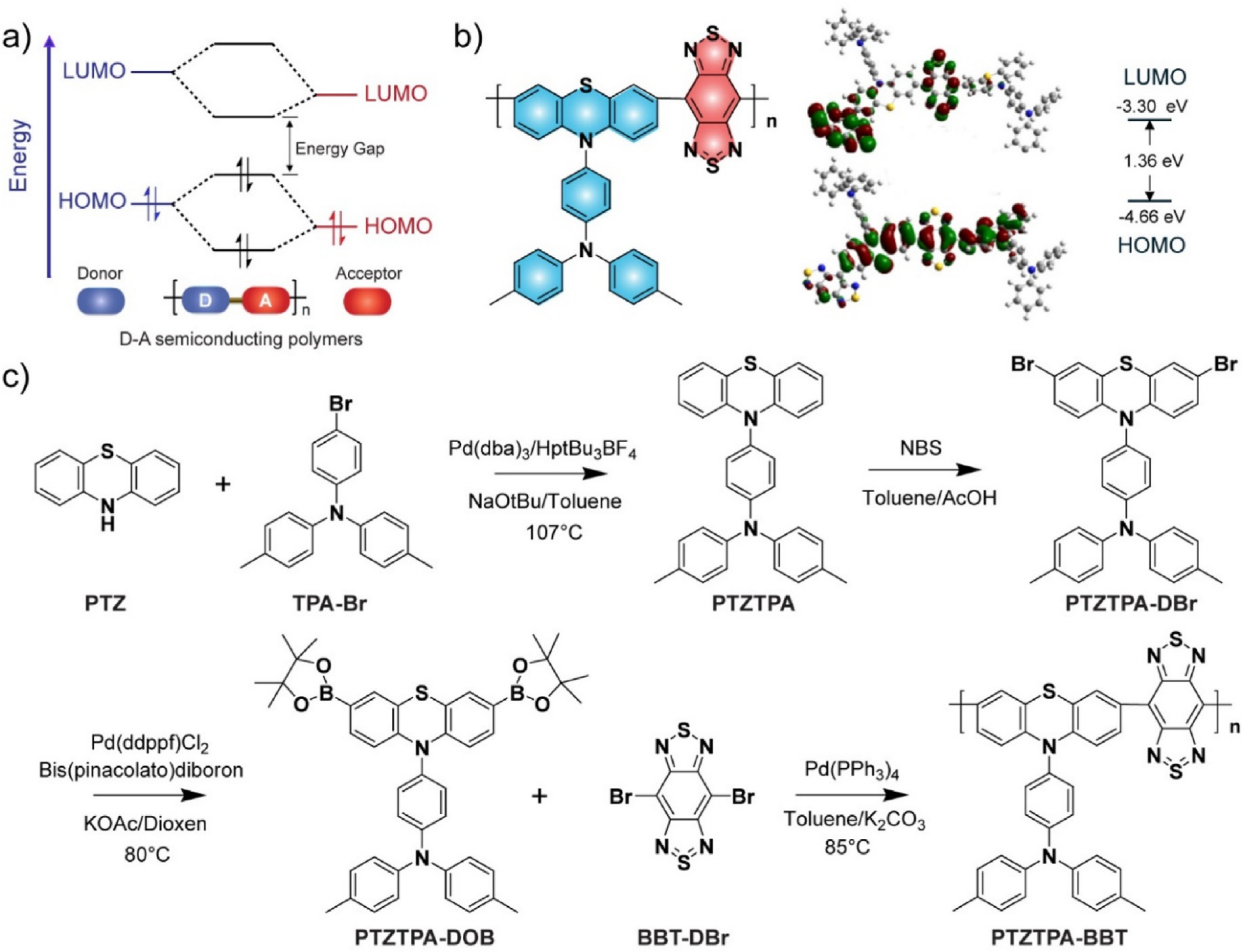
Design and synthesis of NIR-II semiconducting polymers. (a) The energy level diagram shows the mechanism of the reduction of HOMO and LUMO gaps caused by the interactions between molecular orbitals of donor and acceptor units in D-A semiconducting polymers. (b) Chemical structure and optimized molecular orbitals of PTZTPA-BBT polymer. (c) Synthesis route of the semiconductor polymer PTZTPA-BBT.

There is a great interest in developing novel semiconducting polymers for biomedical applications [45,46]. The band gap of semiconducting polymers can be effectively tuned by a design approach through donor-acceptor (D-A) interactions (Fig. 2a). In the classical D-A structure, the highest occupied molecular orbital (HOMO) of the donor unit and the lowest unoccupied molecular orbital (LUMO) of the acceptor unit are mainly responsible for the location of the frontier orbitals. Therefore, a narrow band gap between the HOMO and the LUMO can be achieved by using strong donor and acceptor units. Here we choose triphenylamine (TPA) functionalized phenothiazine (PTZ) as the donor due to its excellent electron donating ability and outstanding nonplanar structure to enhance the fluorescence. Furthermore, we attempted to drive the fluorescence emission of the semiconducting polymer to the NIR-II window by using the extremely strong acceptor benzothiazole (BBT).

Density functional theory (DFT) calculations were used to predict the geometric configuration and optical band gaps of the D-A semiconducting polymer PTZTPA-BBT (Fig. 2b). The LUMO of PTZTPA-BBT was mainly localized on the BBT acceptor unit, whereas the HOMO was distributed along the whole PTZTPA-BBT backbone, indicating strong intramolecular charge transfer within the PTZTPA-BBT polymer backbone to result in long-wavelength optical activity. The corresponding S1/T1 values showed that the PTZTPA-BBT polymers possessed low band gap of 1.36 ​eV. According to the molecular engineering strategies, the synthetic routes of PTZTPA-BBT were designed and carried out (Fig. 2c). The TPA group was attached to the PTZ via nucleophilic substitution, and the resulted intermediate PTZTPA (Fig. S1) was brominated (PTZTPA-DBr, Fig. S2) and boronized (PTZTPA-DOB, Fig. S3) by the Miyaura reaction. Finally, PTZTPA-DOB and brominated BBT (BBT-Br) are polymerized through the Suzuki reaction. Detailed synthesis information was provided in Supporting Information. Gel permeation chromatography (GPC) measurement was performed to quantify number-average molecular weight (*M*_*n*_) of PTZTPA-BBT (4900 ​Da) with a polymer dispersity index (PDI) of 1.56.

### 2.2 Preparation and Characterization of Pdot-C6

**Fig. 3:**
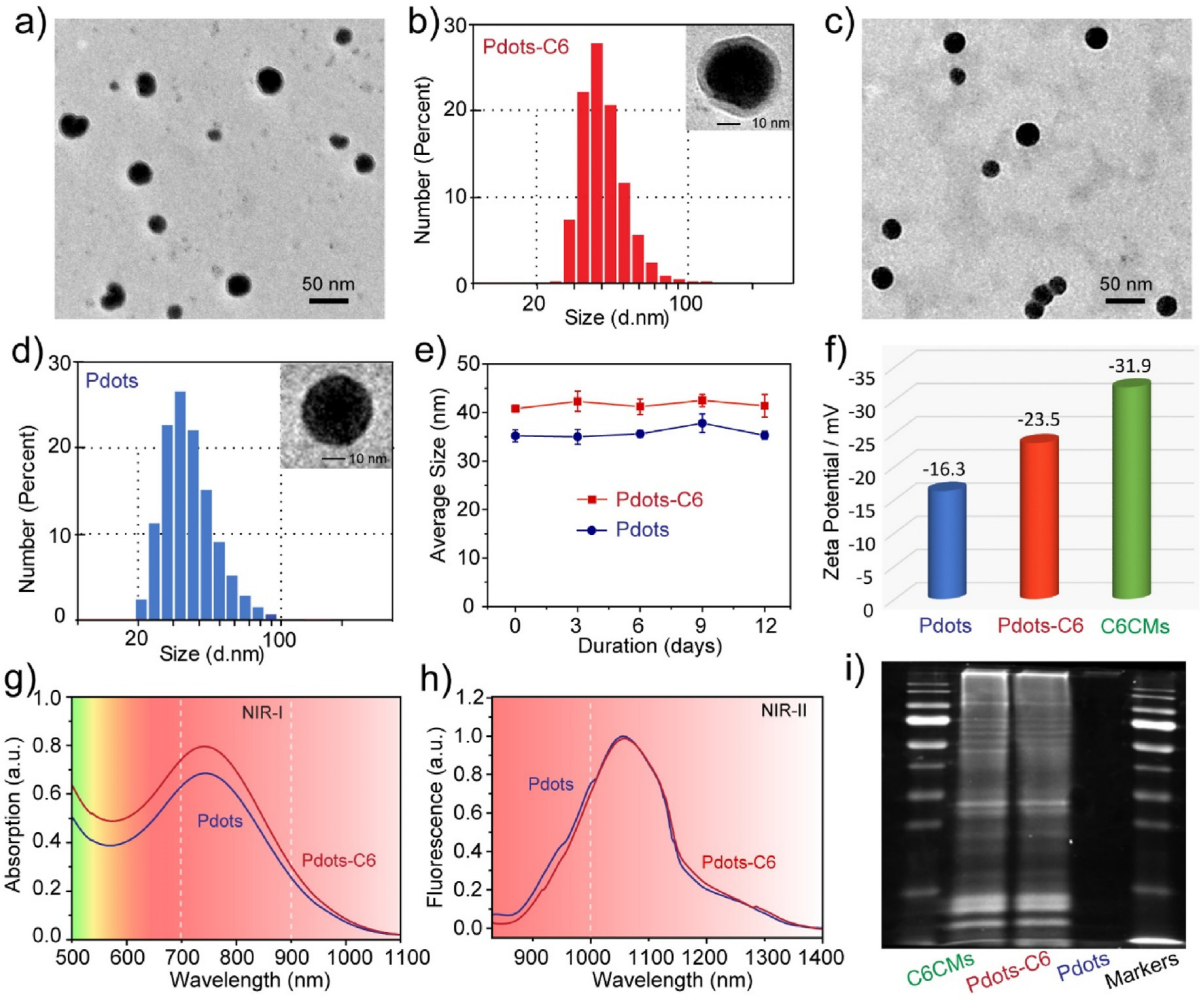
Characterization of Pdot-C6. (a) The TEM image of Pdots-C6. (b) The hydrodynamic diameter and TEM image of Pdots-C6. (c) The TEM image of Pdots. (d) The hydrodynamic diameter and TEM image of Pdots. (e) The hydrodynamic diameters of Pdots and Pdots-C6 versus the storage time at room temperature. Bars represent the mean ​± ​SD (*n* ​= ​3). (f) Zeta potential of Pdots, Pdots-C6 and C6 cell membranes (C6CMs). (g) Absorption spectra of Pdots and Pdots-C6. (h) Fluorescence spectra of Pdots and Pdots-C6. (i) SDS-PAGE protein analysis of C6CMs, Pdots-C6, Pdots, and protein markers.

Several methods are available right now for preparing membrane-coated nanoparticles [47]. The Pdots-C6 were fabricated according to previous approach [32,48], in which the Pdots core and purified C6 cell membranes were coextruded through a porous membrane. The preparation method consists of the following steps. First, semiconducting polymer PTZTPA-BBT and co-polymer polystyrene graft ethylene oxide functionalized with carboxylic end group (PS-PEG-COOH) were utilized to produce Pdots through the nanoprecipitation method. In addition, the C6 cell membrane components were extracted from harvested C6 glioma cells by emptying the cells. Further, the purified C6 membrane protein was linked to the surface of PTZTPA-BBT Pdots by extrusion using the filter membrane at 200 ​nm. The mechanical force provided by the extrusion drives Pdots smaller than 200 ​nm in size to cross the lipid bilayer, resulting in vesicle-particle fusion [49]. The issue on incomplete particle coating during liposome-particle fusion can be resolved by using repeatedly passing through the extruder [50]. Transmission electron microscopy (TEM) imaging and dynamic light scattering (DLS) results demonstrated that Pdots-C6 had the spherical shape and a typical core-shell structure (Fig. 3a) with an average particle size of 40.8 ​nm (Fig. 3b), which was slightly larger than that of the parental Pdots (35.2 ​nm) (Fig. 3c and d). It was also discovered that the obtained Pdots and Pdots-C6 remained stable for more than 10 days, demonstrating the feasibility of the subsequent experiments (Fig. 3e). After Pdots were coated with C6 cell membranes (C6CMs), the zeta potential of Pdots-C6 also increased from −16.3 to −23.5 ​mV (Fig. 3f). The TEM, DLS and zeta-potential analysis results consistently illustrated that C6CMs were successfully translocated to the Pdots surface.

Besides, the optical properties of PTZTPA-BBT Pdots and Pdots-C6 were respectively examined by using the absorption and NIR emission spectrum. PTZTPA-BBT Pdots and Pdots-C6 showed almost the same absorption spectra with a peak at 745 ​nm (Fig. 3g). Meanwhile, the fluorescence spectrum of both Pdots and Pdots-C6 exhibited a strong emission peak at 1055 ​nm upon excitation at 808 ​nm (Fig. 3h). The NIR-II fluorescence quantum yield (QY) of PTZTPA-BBT Pdots was determined to be 0.6% under 808 ​nm excitation using IR-26 as a reference [41]. Further, the protein profiles of Pdots, C6CMs, and Pdots-C6 were analyzed by sodium dodecyl sulfate–polyacrylamide gel electrophoresis (SDS-PAGE). As shown in Fig. 3i, the composition of C6CMs proteins was mostly retained in Pdots-C6, and no protein signal was detected in Pdots. This findings also indicated that the Pdots core was successfully coated by C6CMs and the Pdots-C6 preserved the membrane proteins of original C6 cells.

### 2.3 Biocompatibility and Anti-phagocytic characteristics of Pdots-C6

**Fig. 4:**
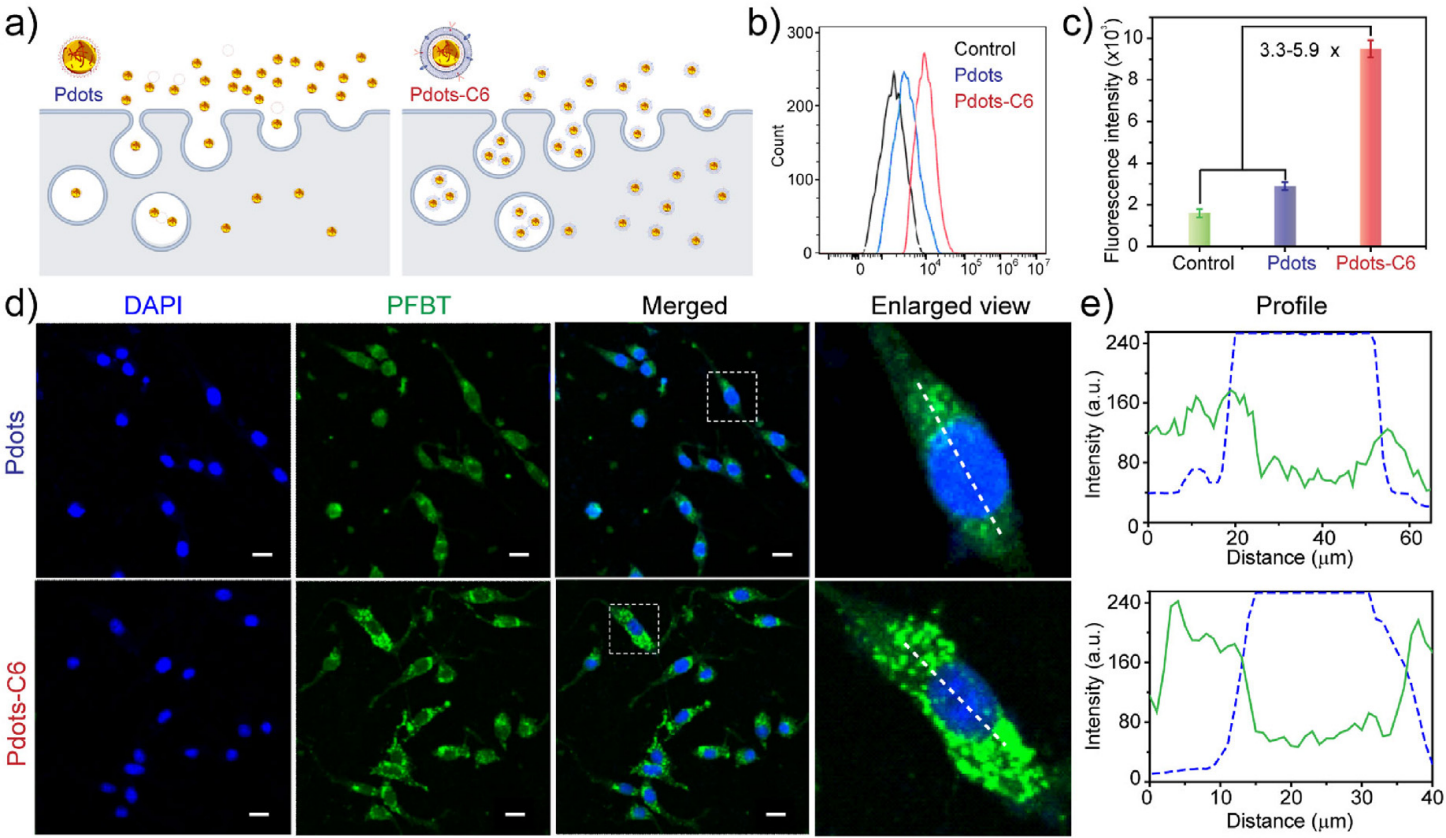
Anti-phagocytic characteristics of Pdots-C6. (a) Schematic diagram of cell uptake difference between Pdots and Pdots-C6. (b) Intracellular fluorescence intensity distribution of C6 cells analyzed by flow cytometry after treated with Pdots and Pdots-C6. (c) The mean fluorescence intensity values of C6 cells after incubation with Pdots and Pdots-C6. Bars represent the mean ​± ​SD (*n* ​= ​3). (d) Fluorescence microscope images of C6 cells treated with Pdots and Pdots-C6, respectively. Scale bar ​= ​20 ​μm. (e) Intracellular fluorescence intensity of C6 cells analyzed by fluorescence microscope images after treated with Pdots and Pdots-C6.

The in vitro biocompatibility and cellular uptake of fabricated Pdots and Pdots-C6 were respectively inspected. Exogenous nanoprobes entering the cells can be recognized by the immune system and cleared by macrophages, resulting in limited diagnostic and therapeutic efficiency. In particular, membrane proteins on the surface of homologous C6CMs can actively signal “don't eat me”to macrophages [51], preventing themselves from being cleared (Fig. 4a).

In this study, a polymer poly [(9,9-dioctylfluorenyl-2,7-diyl)-co-(1,4-benzo-{2,1′,3}-thiadazole)] (PFBT) was used to prepare two nanoprobes (PTZTPA-BBT:PFBT Pdots and PTZTPA-BBT:PFBT Pdots-C6), which emitted green fluorescence (Fig. S4). The size of as-prepared PTZTPA-BBT:PFBT Pdots-C6 showed no obvious change as compared to that of PTZTPA-BBT Pdots-C6 before blending with PFBT (Fig. S5). First, the in vitro cell cytotoxicity of Pdots and Pdots-C6 was also respectively inspected by using C6 cells with various concentrations of Pdots (0, 3, 6, 12, 25, 50, and 100 ​μg/mL) and CCK-8 assays (Fig. S6). Then PTZTPA-BBT:PFBT Pdots and PTZTPA-BBT:PFBT Pdots-C6 were respectively incubated with C6 cells for 6 ​h. Flow cytometry was performed to quantify the uptake efficiency of PTZTPA-BBT:PFBT Pdots and PTZTPA-BBT:PFBT Pdots-C6 in C6 cells, respectively (Fig. 4b). The fluorescence intensity of the PTZTPA-BBT: PFBT Pdots-C6 group was about 3.3 times higher than that of the pure PTZTPA-BBT:PFBT Pdots group (Fig. 4c). The high binding efficiency was largely due to the internalized homologous targeting ability of PTZTPA-BBT: PFBT Pdots-C6 group. For the qualitative uptake analysis, the cell nucleus was labeled with 4′,6-diamidino-2-phenylindole (DAPI), which emitted blue fluorescence. Since PFBT emitted green fluorescence, both PTZTPA-BBT: PFBT Pdots and PTZTPA-BBT: PFBT Pdots-C6 were able to emit green fluorescence as well. In particular, a merged view of the two channels was also offered for comparison (Fig. 4d). The imaging results demonstrated that stronger green fluorescence was detected in the PTZTPA-BBT: PFBT Pdots-C6 group as compared to that from pure PTZTPA-BBT: PFBT Pdots. Intracellular fluorescence intensity of C6 cells was measured by using fluorescence microscope images after treated with Pdots and Pdots-C6, respectively (Fig. 4e). The analysis results indicated that C6 cells have a significantly high tendency to uptake C6CMs coated Pdots, demonstrating the targeting capability of the cell membrane coating strategy.

### 2.4 In vivo NIR-II imaging of Brian tumor

**Fig. 5:**
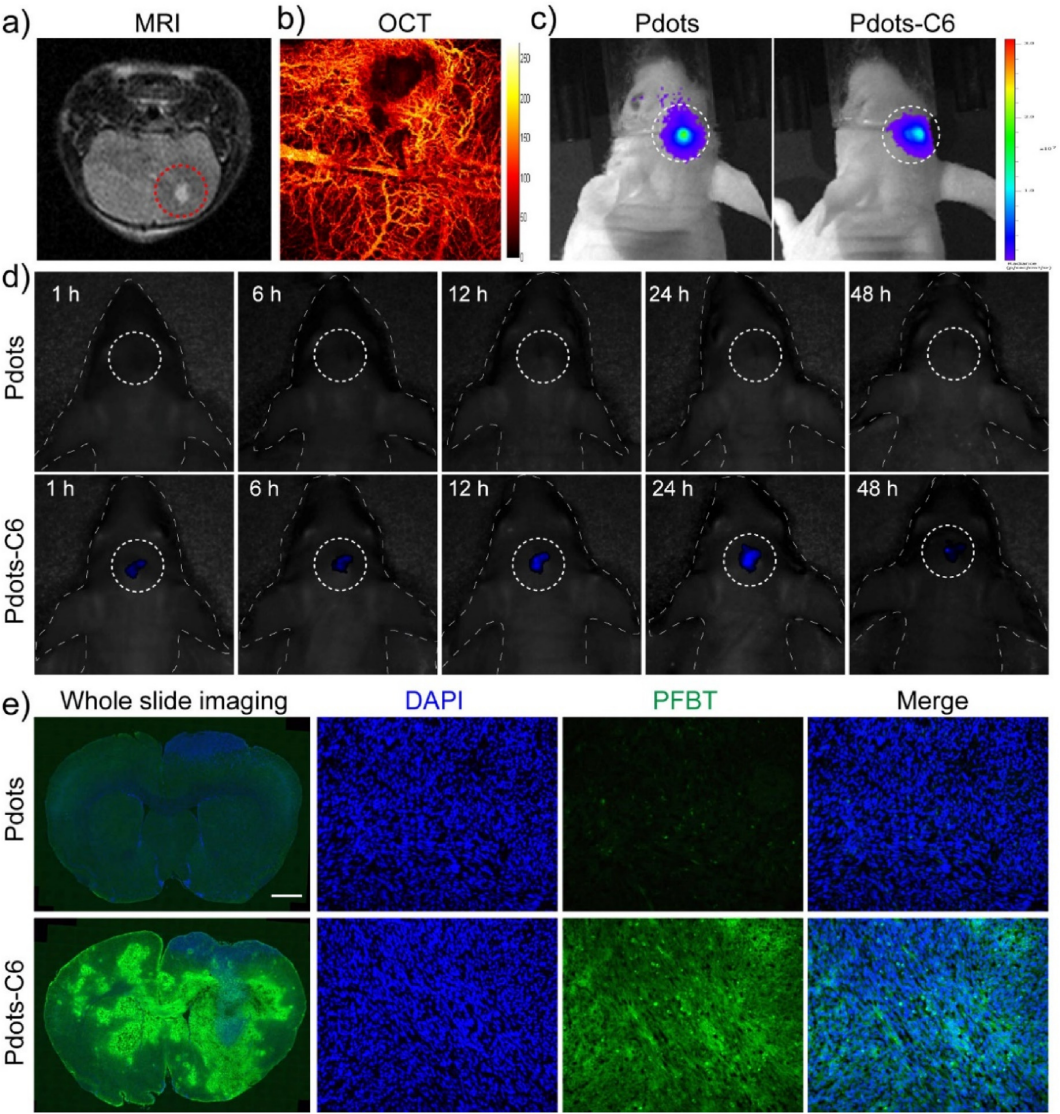
(a) Coronal section view by MRI of glioma-bearing mouse. (b) In vivo OCT image of glioma-bearing mouse. (c) In vivo fluorescence images of glioma-bearing mice. (d) In vivo NIR-II fluorescence imaging of glioma with Pdots and Pdots-C6 administration at different time points post-injection. (e) Brain tissue section 24 h post-injection. Green: PFBT; blue: cell nuclei stained with DAPI. Scale bar ​= ​1 ​mm. (For interpretation of the references to color in this figure legend, the reader is referred to the Web version of this article.)

Inspired by the excellent biocompatibility and active-targeting ability of Pdots-C6 from in vitro test results, in vivo experiments were performed using brain tumor-bearing BALB/c nude mice model. Luciferase-labeled C6 glioma cells (C6-Luc) were injected into the mouse striatum to construct glioma orthotopic implantation tumor model. After the mice were fixed on a digital stereotaxic instrument, cells (5 ​× ​10^5^ ​cells/mouse) were seeded into the hippocampus. The contact surface between the syringe of brain stereotaxic instrument and brain tissues was zero interface, and the needle injection depth was 4 ​mm. After 5 ​min, the depth was adjusted to 3.5 ​mm and cells were injected immediately. After inoculation, the skull was sealed with bone wax while the scalp was sutured. The use of C6-Luc to construct the tumor model allowed us to assess the viability of glioma cells by fluorescence imaging. Besides, MRI and optical coherence tomography (OCT) were performed to monitor the growth of intracranial glioma cells. MRI demonstrated that the depth of glioma was about 3.55 ​mm after 8 days of tumor cell inoculation (Fig. 5a), whereas the blood vascular structures in glioma microenvironments were able to be visualized by OCT (Fig. 5b). The MRI/OCT multimodal imaging results demonstrated that the brain tumor model was successfully constructed.

In addition, NIR-II fluorescence imaging was conducted to access the targeting ability of Pdots and Pdots-C6 in living body, respectively. Due to reporter-gene labeled C6 glioma cells labeled by luciferase, fluorescence signal intensity was able to be inspected in the tumor site of the orthotopic glioma mouse (Fig. 5c). The mice were randomly divided into two groups (*n* ​= ​3 mice for each group): 1) the pure Pdots control group, and 2) the Pdots-C6 group. After tail-vein injection of Pdots or Pdots-C6, the fluorescent signals from the two groups of mice were monitored at 1, 6, 12, 24, and 48 ​h post-injection, respectively (Fig. 5d). Substantial fluorescence was clearly detected in the brains of mice for the Pdots-C6 group. However, this is not the case for pure Pdots group, in which no obvious fluorescence signals were identified. More importantly, the Pdots-C6 group exhibited an improved brain accumulation rate and enhanced fluorescence signals (Fig. 5e). This pilot results demonstrated that the Pdots-C6 group exhibited an improved brain accumulation rate as compared to the pure Pdots group, due to the better tumor targeting and BBB crossing ability of C6CMs.

## 3 Conclusion

In summary, we have successfully fused natural C6 membranes onto the surfaces of the Pdots, resulting in Pdots-C6 that perfectly integrates the biomimetic function, high homotypic binding and glioma-targeting, and BBB crossing abilities. Due to the improved delivery of Pdots across BBB, Pdots-C6 was constructed for enhanced NIR-II fluorescence imaging of glioma through homologous targeting mechanism. Pdots-C6 mediated NIR-II fluorescence imaging significantly improves imaging capabilities as compared to Pdots, leading to high-accuracy glioma detection. These findings provide insights for the development of a biomimetic nanoplatform for the precise diagnosis of glioma.

## 4 Experimental section

### 4.1 Theoretical calculation

The DFT method was employed to calculate the electronic properties of the synthetic polymers. To reduce the computational cost, the monomer and oligomer of the polymers were chosen as the calculating model. The ground-state (S_0_) geometries of four polymer models were completely optimized using the functional B3LYP combined with the 6-31G(d) basis set. The HOMO energy, the LUMO energy and the energy gap were calculated to investigate the electronic and optical properties of polymers from a theoretical perspective.

### 4.2 Preparation of C6 glioma cell membranes

C6 membrane proteins were firstly processed with direct extrusion and then centrifuged to extract the cell membranes [52,53]. Briefly, C6 cells were cultured with complete culture medium in a 175T cell culture flask and subsequently grown into about 80% at 37 ​°C. Further, C6 cells (2.5 ​× ​10^7^ ​cells/mL) were harvested and resuspended in ice-cold tris-magnesium buffer for 30 ​min at 4 ​°C. To disrupt the cell's structure, a mini-extruder with a 0.4 ​μm polyester porous membrane filter was carried out to produce cell homogenate. Finally, the cell homogenate was added into the ice-cold TM-buffer with 0.25 ​M of sucrose. In particular, the mixture was centrifuged for 10 ​min at 2000×*g* and 4 ​°C to remove the intracellular content. As a result, the cell membrane components were collected by centrifugation at 3000×*g* and 4 ​°C for 30 ​min. And the protein content was determined by bicinchoninic acid (BCA) protein assay.

### 4.3 Preparation of Pdots-C6

PTZTPA-BBT Pdots were synthesized based on the previously reported nanoprecipitation method with a simple sonication [54]. In a typical reprecipitation, conjugated polymer PTZTPA-BBT (100 ​μg ​mL^−1^) and functional polymer PS-PEG-COOH (30 ​μg ​mL^−1^) were dissolved in fresh THF. And then the mixture solution (3 ​mL, 100 ​μg ​mL^−1^ PTZTPA-BBT) was rapidly poured into 10 ​mL Milli-Q (10 ​mL) water under vigorous ultrasonication for 3 ​min. Further, the resultant Pdots suspension was heated to get rid of the THF solution with nitrogen gas protection. The Pdots suspension was filtrated using a 0.22-μm membrane filter to remove the aggregates Pdots. Pdots-C6 were constructed by coextruding of a purified C6 membrane proteins and Pdots together through the filter membrane at 200 ​nm.

### 4.4 Characterization of Pdots-C6

The particle size (diameter, nm) and surface charge (zeta potential, mV) of Pdots and Pdot-C6 were respectively measured by using DLS on a Zetasizer Nano ZS (Nano ZS90, Ltd.). The absorbance was recorded on the UV-1800 spectrophotometer (Shimadzu, Japan), whereas the fluorescent spectroscopy was measured with a fluorescence spectrophotometer (FluoroMax-4, Horiba, Japan). The protein profiles of C6CMs, Pdots, and Pdots-C6 were obtained using SDS-PAGE protein analysis. Flow cytometry analysis was performed on a BD Biosciences FACS Calibur HTS. To analyze the frequency, a total of 20, 000 ​cells were counted.

## CRediT authorship contribution statement

**Xiaoju Men:** Methodology, Formal analysis, Investigation, Visualization, Writing – original draft, Funding acquisition. **Xiaorui Geng:** Validation, Formal analysis, Investigation, Visualization. **Zhe Zhang:** Formal analysis, Investigation, Resources, Visualization, Validation, Data curation. **Haobin Chen:** Conceptualization, Methodology, Visualization, Writing – review & editing. **Meng Du:** Methodology. **Zhiyi Chen:** Methodology. **Gang Liu:** Methodology. **Changfeng Wu:** Conceptualization, Methodology, Funding acquisition. **Zhen Yuan:** Supervision, Project administration, Writing – review & editing, Funding acquisition.

## Declaration of competing interest

The authors declare that they have no known competing financial interests or personal relationships that could have appeared to influence the work reported in this paper.

## References

[1] Baratta M.G. (2018). Getting to the brain. Nat. Nanotechnol..

[2] Touat M., Li Y.Y., Boynton A.N., Spurr L.F., Iorgulescu J.B., Bohrson C.L., Cortes-Ciriano I., Birzu C., Geduldig J.E., Pelton K. (2020). Mechanisms and therapeutic implications of hypermutation in gliomas. Nature.

[3] DeAngelis L.M. (2001). Brain tumors. N. Engl. J. Med..

[4] Bahadure N.B., Ray A.K., Thethi H.P. (2017). Image analysis for MRI based brain tumor detection and feature extraction using biologically inspired BWT and SVM. Int. J. Biomed. Imag..

[5] Bauer S., Wiest R., Nolte L.-P., Reyes M. (2013). A survey of MRI-based medical image analysis for brain tumor studies. Phys. Med. Biol..

[6] Overcast W.B., Davis K.M., Ho C.Y., Hutchins G.D., Green M.A., Graner B.D., Veronesi M.C. (2021). Advanced imaging techniques for neuro-oncologic tumor diagnosis, with an emphasis on PET-MRI imaging of malignant brain tumors. Curr. Oncol. Rep..

[7] Chen D., Liu Y., Zhang Z., Liu Z., Fang X., He S., Wu C. (2020). NIR-II fluorescence imaging reveals bone marrow retention of small polymer nanoparticles. Nano Lett..

[8] Tang Y., Li Y., Hu X., Zhao H., Ji Y., Chen L., Hu W., Zhang W., Li X., Lu X. (2018). Dual lock-and-key”-controlled nanoprobes for ultrahigh specific fluorescence imaging in the second near-infrared window. Adv. Mater..

[9] Huang J., Pu K. (2020). Activatable molecular probes for second near-infrared fluorescence, chemiluminescence, and photoacoustic imaging. Angew. Chem. Int. Ed..

[10] Hu Z., Fang C., Li B., Zhang Z., Cao C., Cai M., Su S., Sun X., Shi X., Li C. (2020). First-in-human liver-tumour surgery guided by multispectral fluorescence imaging in the visible and near-infrared-I/II windows. Nat. Biomed. Eng..

[11] Smith A.M., Mancini M.C., Nie S. (2009). Second window for in vivo imaging. Nat. Nanotechnol..

[12] Weissleder R. (2001). A clearer vision for in vivo imaging. Nat. Biotechnol..

[13] Kuang Y., Liu N., Ye S., Li X., Chen X., Qi L., Zhu P., Liu R., Wu X. (2022). Ce doped polyaniline nanoparticles for absorption and photoacoustic imaging response to GSH in vitro and in vivo. Bioact. Mater..

[14] Liu M.H., Zhang Z., Yang Y.C., Chan Y.H. (2021). Polymethine-based semiconducting polymer dots with narrow-band emission and absorption/emission maxima at NIR-II for bioimaging. Angew. Chem. Int. Ed..

[15] Li C., Luo Z., Yang L., Chen J., Cheng K., Xue Y., Liu G., Luo X., Wu F. (2022). Self-assembled porphyrin polymer nanoparticles with NIR-II emission and highly efficient photothermal performance in cancer therapy, Mater. Today Bio.

[16] Huang J., Xie C., Zhang X., Jiang Y., Li J., Fan Q., Pu K. (2019). Renal-clearable molecular semiconductor for second near-infrared fluorescence imaging of kidney dysfunction. Angew. Chem. Int. Ed..

[17] Dal Magro R., Ornaghi F., Cambianica I., Beretta S., Re F., Musicanti C., Rigolio R., Donzelli E., Canta A., Ballarini E. (2017). ApoE-modified solid lipid nanoparticles: a feasible strategy to cross the blood-brain barrier. J. Contr. Release.

[18] Gonzalez-Carter D., Liu X., Tockary T.A., Dirisala A., Toh K., Anraku Y., Kataoka K. (2020). Targeting nanoparticles to the brain by exploiting the blood–brain barrier impermeability to selectively label the brain endothelium. Proc. Natl. Acad. Sci. U.S.A..

[19] Dash P., Piras A.M., Dash M. (2020). Cell membrane coated nanocarriers-an efficient biomimetic platform for targeted therapy. J. Contr. Release.

[20] Chen H.-Y., Deng J., Wang Y., Wu C.-Q., Li X., Dai H.-W. (2020). Hybrid cell membrane-coated nanoparticles: a multifunctional biomimetic platform for cancer diagnosis and therapy. Acta Biomater..

[21] Li J., Zhen X., Lyu Y., Jiang Y., Huang J., Pu K. (2018). Cell membrane coated semiconducting polymer nanoparticles for enhanced multimodal cancer phototheranostics. ACS Nano.

[22] Luk B.T., Zhang L. (2015). Cell membrane-camouflaged nanoparticles for drug delivery. J. Contr. Release.

[23] Angsantikul P., Fang R.H., Zhang L. (2017). Toxoid vaccination against bacterial infection using cell membrane-coated nanoparticles. Bioconjugate Chem..

[24] Gong H., Zhang Q., Komarla A., Wang S., Duan Y., Zhou Z., Chen F., Fang R.H., Xu S., Gao W. (2021). Nanomaterial biointerfacing via mitochondrial membrane coating for targeted detoxification and molecular detection. Nano Lett..

[25] Ai X., Wang S., Duan Y., Zhang Q., Chen M.S., Gao W., Zhang L. (2020). Emerging approaches to functionalizing cell membrane-coated nanoparticles. Biochemistry.

[26] Vijayan V., Uthaman S., Park I.-K. (2018). Cell membrane-camouflaged nanoparticles: a promising biomimetic strategy for cancer theragnostics. Polymers.

[27] Zhang X., He S., Ding B., Qu C., Zhang Q., Chen H., Sun Y., Fang H., Long Y., Zhang R., Lan X., Cheng Z. (2020). Cancer cell membrane-coated rare earth doped nanoparticles for tumor surgery navigation in NIR-II imaging window. Chem. Eng. J..

[28] Yurkin S.T., Wang Z. (2017). Cell membrane-derived nanoparticles: emerging clinical opportunities for targeted drug delivery. Nanomedicine.

[29] Rao L., Bu L.L., Xu J.H., Cai B., Yu G.T., Yu X., He Z., Huang Q., Li A., Guo S.S., Zhang W.-F., Liu W., Sun Z.-J., Wang H., Wang T.-H., Zhao X.-Z. (2015). Red blood cell membrane as a biomimetic nanocoating for prolonged circulation time and reduced accelerated blood clearance. Small.

[30] Xia Q., Zhang Y., Li Z., Hou X., Feng N. (2019). Red blood cell membrane-camouflaged nanoparticles: a novel drug delivery system for antitumor application. Acta Pharm. Sin. B.

[31] Zhao Y.-Z., Shen B.-X., Li X.-Z., Tong M.-Q., Xue P.-P., Chen R., Yao Q., Chen B., Xiao J., Xu H.-L. (2020). Tumor cellular membrane camouflaged liposomes as a non-invasive vehicle for genes: specific targeting toward homologous gliomas and traversing the blood–brain barrier. Nanoscale.

[32] Deng G., Peng X., Sun Z., Zheng W., Yu J., Du L., Chen H., Gong P., Zhang P., Cai L. (2020). Natural-killer-cell-inspired nanorobots with aggregation-induced emission characteristics for near-infrared-II fluorescence-guided glioma theranostics. ACS Nano.

[33] Beh C.Y., Prajnamitra R.P., Chen L.-L., Hsieh P.C.-H. (2021). Advances in biomimetic nanoparticles for targeted cancer therapy and diagnosis. Molecules.

[34] Gao D., Guo X., Zhang X., Chen S., Wang Y., Chen T., Huang G., Gao Y., Tian Z., Yang Z. (2020). Multifunctional phototheranostic nanomedicine for cancer imaging and treatment. Mater. Today Bio..

[35] Chen H., Yu J., Zhang J., Sun K., Ding Z., Jiang Y., Hu Q., Wu C., Chiu D.T. (2021). Monitoring metabolites using an NAD (P) H-sensitive polymer dot and a metabolite-specific enzyme. Angew. Chem. Int. Ed..

[36] Men X., Wang F., Chen H., Liu Y., Men X., Yuan Y., Zhang Z., Gao D., Wu C., Yuan Z. (2020). Ultrasmall semiconducting polymer dots with rapid clearance for second near-infrared photoacoustic imaging and photothermal cancer therapy. Adv. Funct. Mater..

[37] Chen H., Fang X., Jin Y., Hu X., Yin M., Men X., Chen N., Fan C., Chiu D.T., Wan Y. (2018). Semiconducting polymer nanocavities: porogenic synthesis, tunable host–guest interactions, and enhanced drug/siRNA delivery. Small.

[38] Chen Y., Sun B., Jiang X., Yuan Z., Chen S., Sun P., Fan Q., Huang W. (2021). Double-acceptor conjugated polymers for NIR-II fluorescence imaging and NIR-II photothermal therapy applications. J. Mater. Chem. B.

[39] Zhang W., Huang T., Li J., Sun P., Wang Y., Shi W., Han W., Wang W., Fan Q., Huang W. (2019). Facial control intramolecular charge transfer of quinoid conjugated polymers for efficient in vivo NIR-II imaging. ACS Appl. Mater. Interfaces.

[40] Liu Y., Liu J., Chen D., Wang X., Zhang Z., Yang Y., Jiang L., Qi W., Ye Z., He S., Liu Q., Xi L., Zou Y., Wu C. (2020). Fluorination enhances NIR-II fluorescence of polymer dots for quantitative brain tumor imaging. Angew. Chem. Int. Ed..

[41] Zhang Z., Fang X., Liu Z., Liu H., Chen D., He S., Zheng J., Yang B., Qin W., Zhang X., Wu C. (2020). Semiconducting polymer dots with dual-enhanced NIR-IIa fluorescence for through-skull mouse-brain imaging. Angew. Chem. Int. Ed..

[42] Julier Z., Park A.J., Briquez P.S., Martino M.M. (2017). Promoting tissue regeneration by modulating the immune system. Acta Biomater..

[43] Varnamkhasti B.S., Jafari S., Taghavi F., Alaei L., Izadi Z., Lotfabadi A., Dehghanian M., Jaymand M., Derakhshankhah H., Saboury A.A. (2020). Cell-penetrating peptides: as a promising theranostics strategy to circumvent the blood-brain barrier for CNS diseases. Curr. Drug Deliv..

[44] Duan Y., Wu M., Hu D., Pan Y., Hu F., Liu X., Thakor N., Ng W.H., Liu X., Sheng Z. (2020). Biomimetic nanocomposites cloaked with bioorthogonally labeled glioblastoma cell membrane for targeted multimodal imaging of brain tumors. Adv. Funct. Mater..

[45] Chen H.B., Wang F., Liu M.Y., Qian M.D., Men X.J., Yao C.F., Xi L., Qin W.P., Qin G.S., Wu C.F. (2019). Near-infrared broadband polymer-dot modulator with high optical nonlinearity for ultrafast pulsed lasers. Laser Photon. Rev..

[46] Chen H., Yu J., Men X., Zhang J., Ding Z., Jiang Y., Wu C., Chiu D.T. (2021). Reversible ratiometric NADH sensing using semiconducting polymer dots. Angew. Chem. Int. Ed..

[47] Fang R.H., Kroll A.V., Gao W., Zhang L. (2018). Cell membrane coating nanotechnology. Adv. Mater..

[48] Deng G., Sun Z., Li S., Peng X., Li W., Zhou L., Ma Y., Gong P., Cai L. (2018). Cell-membrane immunotherapy based on natural killer cell membrane coated nanoparticles for the effective inhibition of primary and abscopal tumor growth. ACS Nano.

[49] Hu C.M.J., Zhang L., Aryal S., Cheung C., Fang R.H., Zhang L. (2011). Erythrocyte membrane-camouflaged polymeric nanoparticles as a biomimetic delivery platform. Proc. Natl. Acad. Sci. U.S.A..

[50] Tanaka M., Sackmann E. (2005). Polymer-supported membranes as models of the cell surface. Nature.

[51] Han X., Shen S., Fan Q., Chen G., Archibong E., Dotti G., Liu Z., Gu Z., Wang C. (2019). Red blood cell–derived nanoerythrosome for antigen delivery with enhanced cancer immunotherapy. Sci. Adv..

[52] Jia Y., Sheng Z., Hu D., Yan F., Zhu M., Gao G., Wang P., Liu X., Wang X., Zheng H. (2018). Highly penetrative liposome nanomedicine generated by a biomimetic strategy for enhanced cancer chemotherapy. Biomater. Sci..

[53] Cao H., Dan Z., He X., Zhang Z., Yu H., Yin Q., Li Y. (2016). Liposomes coated with isolated macrophage membrane can target lung metastasis of breast cancer. ACS Nano.

[54] Chen H., Zhang J., Chang K., Men X., Fang X., Zhou L., Li D., Gao D., Yin S., Zhang X., Yuan Z., Wu C. (2017). Highly absorbing multispectral near-infrared polymer nanoparticles from one conjugated backbone for photoacoustic imaging and photothermal therapy. Biomaterials.

