# Supplementary material for: Biomimetic semiconducting polymer dots for highly specific NIR-II fluorescence imaging of glioma
Source: Mater Today Bio. 2022 Aug 7;16:100383. doi: 10.1016/j.mtbio.2022.100383 (PMC9395678; doi:10.1016/j.mtbio.2022.100383)
Supplement: Multimedia component 1 [file mmc1.pdf]

Supporting Information

Biomimetic Semiconducting Polymer Dots for Highly Specific NIR-II Fluorescence Imaging of Glioma

Xiaoju Men[a,b,c#], Xiaorui Geng[b,c#], Zhe Zhang[d#], Mengze Xu[b,c], Haobin Chen[e], Meng Du[f], Zhiyi Chen[f], Gang Liu[g], Changfeng Wu[d], Zhen Yuan[b,c]*

[a]Hunan Key Laboratory of the Research and Development of Novel Pharmaceutical Preparations, Academician Workstation, Changsha Medical University, Changsha, 410219, China

[b]Faculty of Health Sciences, University of Macau, Macau SAR, 999078, China

[c]Centre for Cognitive and Brain Sciences, University of Macau, Macau SAR, 999078, China

[d]Department of Biomedical Engineering, Southern University of Science and Technology, Shenzhen, Guangdong, China

[e]Department of Biomedical Engineering, School of Basic Medical Sciences, Central South University, Changsha, 410013, China

[f]Institute of Medical Imaging, Hengyang Medical School, University of South China, Hengyang, China

[g]Center for Molecular Imaging and Translational Medicine, Xiamen University, Xiamen, China

* Corresponding author.

E-mail address: zhenyuan@um.edu.mo (Z. Yuan).

1. Materials and Preparation

1.1 Materials:

All materials were obtained from J&K Chemical Ltd. (Beijing, China) and used without further purification unless otherwise indicated. Solvent tetrahydrofuran (THF) (anhydrous, 99.9%) and DAPI were purchased from Sigma-Aldrich (St. Louis, MO, USA). The amphiphilic functional polymer PS-PEG-COOH was ordered from America Dye Source Inc. (Quebec H9X 3T6, Canada). 4,7-dibromobenzo[1,2-c:4,5-c']bis([1,2,5]thiadiazole) was purchased from Derthon Optoelectronic Materials Science & Technology Co., Ltd. (Shenzhen, China). Cell Counting Kit-8 and Western-blotting-related reagents were acquired from Beyotime Biotechnology (Shanghai, China). Ultrapure H_2O (18.25 $M\Omega\cdot cm^{-2}$ at 25°C) was used throughout the study.

1.2 Synthesis of N-(4-(10H-phenothiazine-10-yl)phenyl)-4-methyl-N-(p-tolyl)aniline (PTZTPA)

A flask was charged with Phenothiazine (219 mg, 1.1 mmol), 4-bromo-N,N-di-p-tolylaniline (409 mg, 1.0 mmol), $HPtBu_3BF_4$ (6 mg, 0.02 mmol), NaOtBu (110 mg, 1.1 mmol), $Pd_2(dba)_3$ (9 mg, 0.01 mmol) and toluene (15 mL) before it was sealed with Parafilm. The flask was degassed with three freeze-pump-thaw cycles to remove air. The mixture was heated to 107°C for 40 h and washed with water after cooled down to room temperature. The organic layer was dried with anhydrous Na_2SO_4. The crude products were purified by column chromatography using petroleum ether/dichloromethane (6:1 by volume ratio) as the eluent. A light-yellow solid was obtained. Yields: 223 mg (0.47 mmol,

47%). ^{1}H NMR (400 MHz, $CDCl_3$) δ 7.12 (s, 12H), 6.98 (d, J = 7.3 Hz, 2H), 6.86 (dd, J = 25.8, 18.3 Hz, 4H), 6.31 (d, J = 7.6 Hz, 2H), 2.34 (s, 6H). MS (MALDI-TOF): *m/z* calcd $[M]^+$, 470.63; found, 469.91.

1.3 Synthesis of 4-(3,7-dibromo-10H-phenothiazin-10-yl)-N,N-di-p-tolylaniline (PTZTPA-DBr)

A flask was charged with N-(4-(10H-phenothiazine-10-yl)phenyl)-4-methyl-N-(p-tolyl)aniline (3.00 g, 4.78 mmol), N-Bromosuccinimide (1.78 g, 10 mmol) and mixed solvent (toluene: acetic acid = 15 mL: 50 mL). The mixture was stirred under room temperature and washed with deionized water after 24 h. The organic layer was dried with anhydrous Na_2SO_4. The crude products were purified by column chromatography using petroleum ether as the eluent. A light-yellow solid was obtained. Yields: 35%. ^{1}H NMR (400 MHz, $CDCl_3$) δ 7.28 (dd, J = 74.5, 56.5 Hz, 4H), 7.15 – 7.02 (m, 8H), 7.02 – 6.70 (m, 4H), 6.10 (d, J = 8.9 Hz, 2H), 2.34 (dd, J = 13.6, 7.6 Hz, 6H). MS (MALDI-TOF): *m/z* calcd $[M]^+$, 628.43; found, 627.83.

1.4 Synthesis of 4-(3,7-bis(4,4,5,5-tetramethyl-1,3,2-dioxaborolan-2-yl)-10H-phenothiazin-10-yl)-N,N-di-p-tolylaniline (PTZTPA-DOB)

A flask was charged with 4-(3,7-dibromo-10H-phenothiazin-10-yl)-N,N-di-p-tolylaniline (7.22 g, 10 mmol), Bis(pinacolato)diboron (7.62 g, 30 mmol), KOAc (5.88 g, 60 mmol) and 1,4-Dioxane (30 mL) before it was sealed with Parafilm. The flask was degassed with three freeze-pump-thaw cycles to remove air. The mixture was heated to 80°C for 36 h and washed with water after cooled down to room temperature. The organic layer was dried

with anhydrous Na_2SO_4. The crude products were purified by column chromatography using petroleum ether/dichloromethane (1:1 by volume ratio) as the eluent. A light-yellow solid product was obtained. Yields: 24%. ^1H NMR (400 MHz, $CDCl_3$) δ 7.92 (s, 1H), 7.81 (d, J = 8.3 Hz, 1H), 7.59 – 7.24 (m, 14H), 7.11 (d, J = 30.3 Hz, 1H), 6.23 (d, J = 8.2 Hz, 1H), 2.62 – 2.45 (m, 6H), 1.26 (s, 24H). MS (MALDI-TOF): *m/z* calcd $[M]^+$, 722.56; found, 722.34.

1.5 Synthesis of Polymer PTZTPA-BBT

A flask was charged with PTZTPA-DOB (361 mg, 0.5 mmol), 4,7-dibromobenzo[1,2-c:4,5-c']bis([1,2,5]thiadiazole) (175 mg, 0.5 mmol), K_2CO_3 (aq, 2.0 M, 2 mL), $Pd(PPh_3)_4$ (34 mg, 0.03 mmol) and toluene (20 mL) before it was sealed with Parafilm. The flask was degassed with three freeze-pump-thaw cycles to remove air. The mixture was heated to 80°C for 48 h and washed with water after cooled down to room temperature. The organic layer was concentrated and dropped into methanol (200 mL) under stir. The solution was filtrated, sediment was further purified by Soxhlet extraction with acetone, which gives a deep-green solid product. The polymer's molecular weight was measured by GPC as M_n = 4.9 kDa, polydispersity index (PDI) = 1.56.

2. Experimental Procedures

2.1 Characterization

Hamamatsu photonic multichannel analyzer C10027 equipped with CCD and integrating sphere used for fluorescence quantum yields. Gaussian 09 Version D.01 Package was applied for the Theoretical calculations. The Pdots size and morphology were carried out with a Hitachi H-600 transmission electron microscope (TEM). ^{1}H NMR spectra was recorded on a Varian-300 EX spectrometer and Bruker Avance 400 MHz spectrometer. Chemical shifts were reported in ppm (δ) relative to tetramethylsilane (TMS, $\delta = 0.00$ ppm) as the internal standard. Gel permeation chromatography (GPC) measurement was recorded with a Shimadzu gel permeation chromatography system (Prominence HPLC LC-20A Shimadzu, refractive index detector, polystyrene calibration).

2.2 Cytotoxicity Assays

The activities of C6 cells in vitro were accessed by using Cell Counting Kit-8 assays. C6 cells were seeded in 96-well plates (Costar, Chicago, IL, USA) and grown to about 80%. And then the C6 cells were incubated with PTZTPA-BBT Pdots with different concentrations for 24 h and 48 h, respectively. Further, 10 µL Cell Counting Kit-8 solution was added to each well, in which the samples were incubated for an additional 4 h. Finally, the optical density was measured by using a microplate reader (BioTek Cytation 3).

2.3 Tumor Model

Female BALB/c nude mice (5−6 weeks) were acquired from Beijing Vital River Laboratory Animal Technology Co. Ltd. (China). The protocol for the present study was approved by the Animal Care and Use Committee of the University of Macau (UMARE-033-2020). A glioma model was created by injecting C6 cells into the mouse striatum (5×10^5 cells/mouse). MRI and OCT were used to monitor the growth of intracranial glioma.

2.4 Dada Analysis:

Results were expressed as the Mean ± SD unless stated otherwise. This paper used Image J for imaging analysis and Origin software to draw the histograms.

3. Supplementary Figures

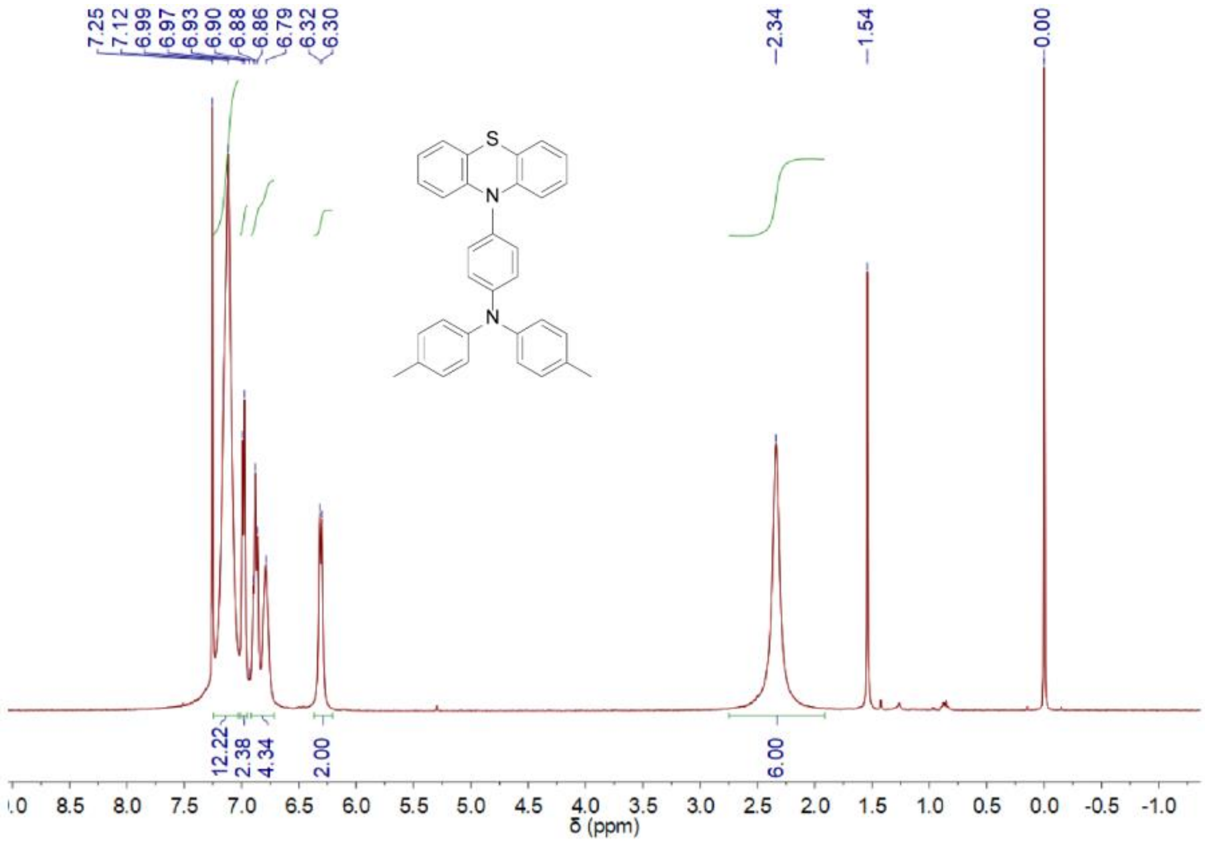

Fig. S1. ^{1}H-NMR ($CDCl_3$) spectra of PTZTPA.

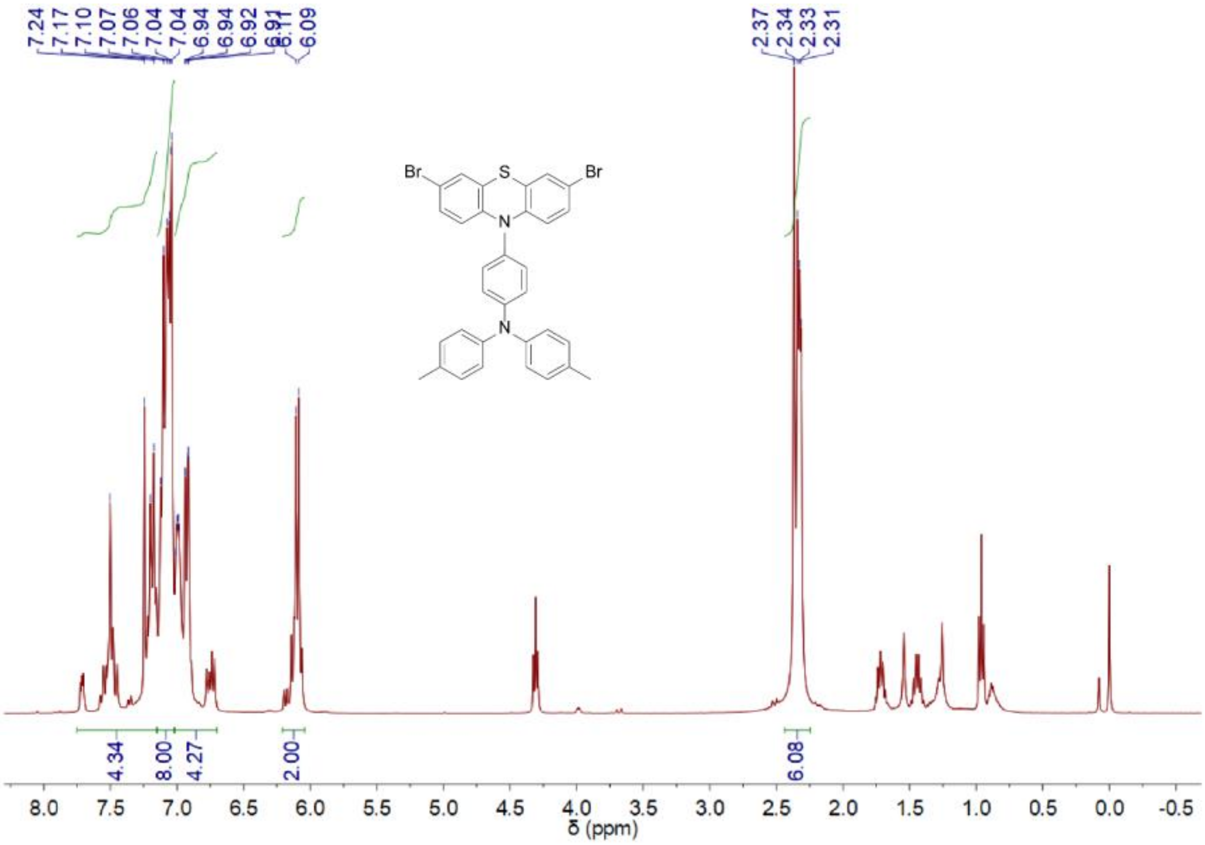

Fig. S2. ^{1}H-NMR ($CDCl_3$) spectra of PTZTPA-DBr.

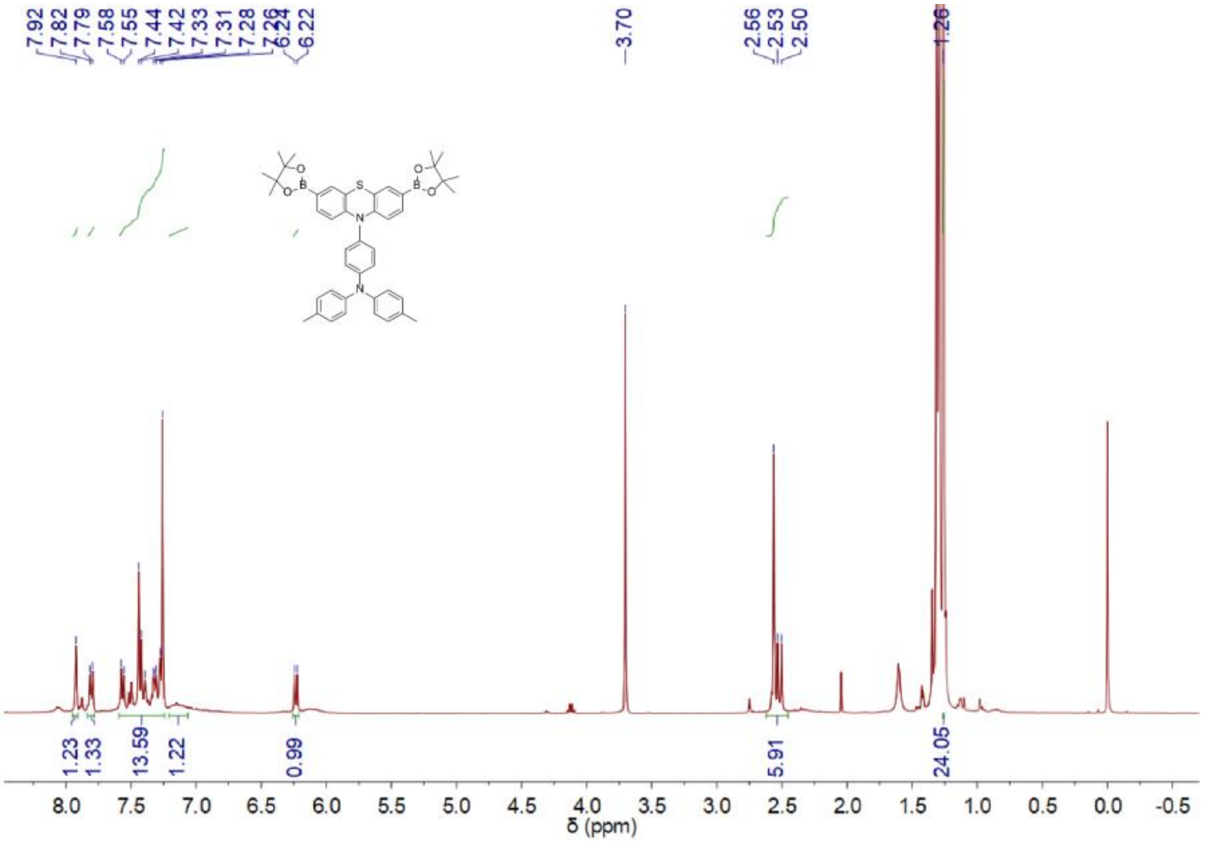

Fig. S3. ^{1}H-NMR ($CDCl_3$) spectra of PTZTPA-DOB.

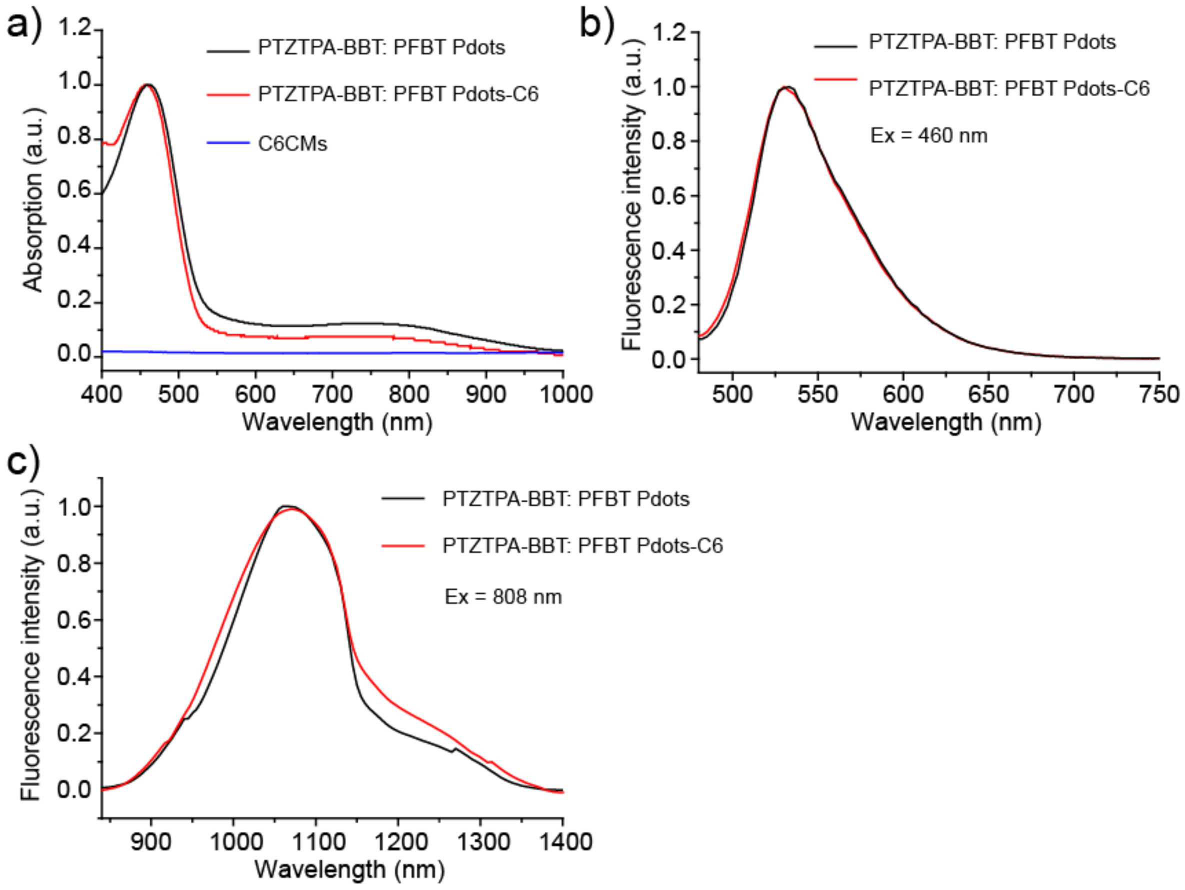

Fig. S4. (a) Absorption spectra of PTZTPA-BBT:PFBT Pdots and PTZTPA-BBT:PFBT Pdots-C6, and C6 cell membranes (C6CMs). (b) Fluorescence spectra of PTZTPA-BBT:PFBT Pdots and PTZTPA-BBT:PFBT Pdots (λ_{ex}=460 nm). (c) Fluorescence spectra of PTZTPA-BBT:PFBT Pdots and PTZTPA-BBT:PFBT Pdots (λ_{ex}=808 nm).

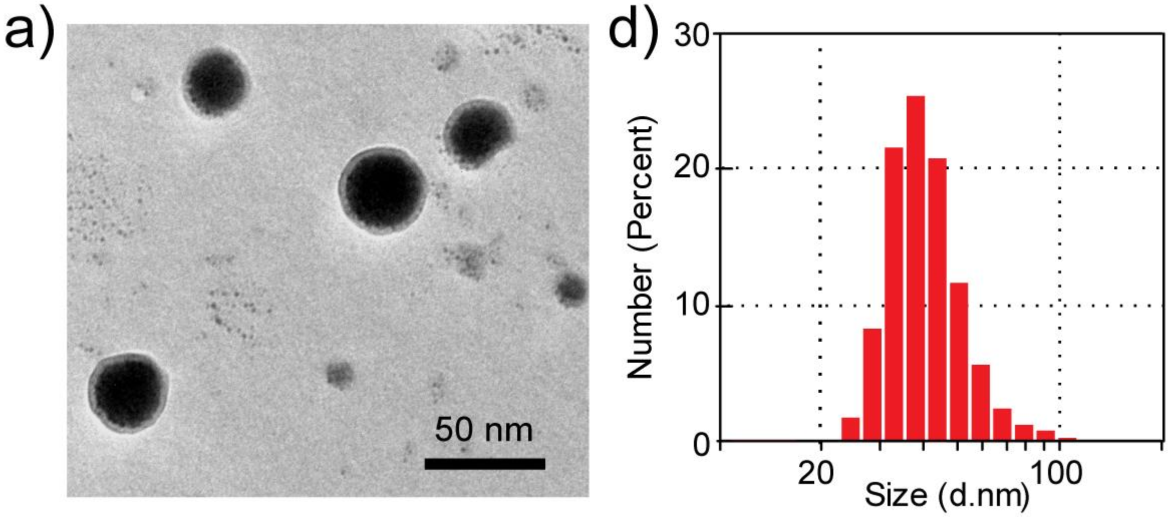

Fig. S5. The hydrodynamic diameter and transmission electron microscopy (TEM) image of PTZTPA-BBT:PFBT Pdots-C6.

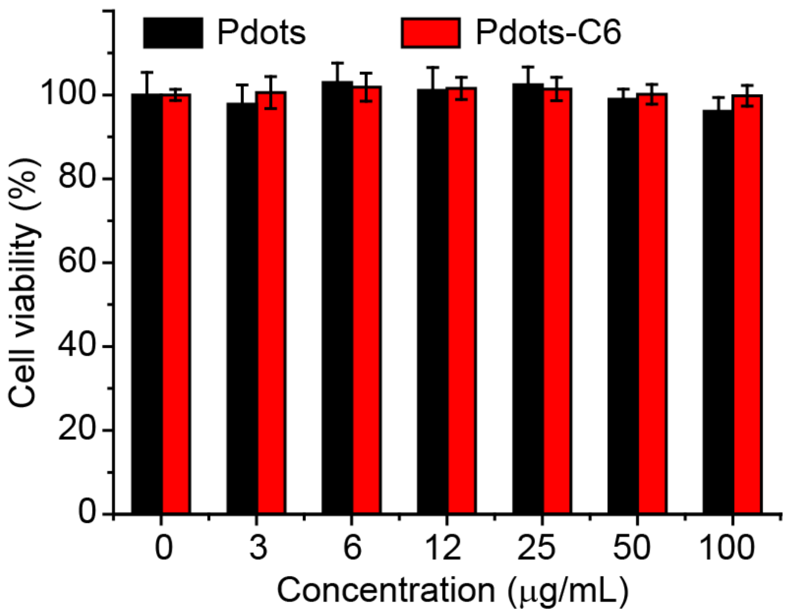

Fig. S6. Cellular viability assays of C6 cells incubated with different concentrations of Pdots and Pdots-C6. Bars represent the mean ± SD (*n* = 6).
